# Effect of MgO Addition on the Mechanical and Dynamic Properties of Zirconia Toughened Alumina (ZTA) Ceramics

**DOI:** 10.3390/ma12152440

**Published:** 2019-07-31

**Authors:** Ali Arab, Zhwan Dilshad Ibrahim Sktani, Qiang Zhou, Zainal Arifin Ahmad, Pengwan Chen

**Affiliations:** 1 State Key Laboratory of Explosion Science and Technology, Beijing Institute of Technology, Beijing 100081, China; 2 Structural Materials Niche Area, School of Materials and Mineral Resources Engineering, Engineering Campus, Universiti Sains Malaysia, Nibong Tebal 14300, Penang, Malaysia; 3 Department of Mechanical and Energy Engineering, Erbil Technical Engineering College, Erbil Polytechnic University, Erbil 44001, Iraq

**Keywords:** dynamic strength, ZTA, MgO, hardness, fracture toughness

## Abstract

Zirconia toughened alumina (ZTA) is a promising advanced ceramic material for a wide range of applications that are subjected to dynamic loading. Therefore, the investigation of dynamic compressive strength, fracture toughness and hardness is essential for ZTA ceramics. However, the relationship between these mechanical properties in ZTA has not yet been established. An example of this relationship is demonstrated using ZTA samples added with MgO prepared through conventional sintering. The microstructure and mechanical properties of ZTA composites were characterized. The hardness of ZTA composites increased for ≤0.7 wt.% MgO due to the pinning effect of MgO and decrease of the porosity in the microstructure. Oppositely, the fracture toughness of ZTA composites continuously decreased due to the size reduction of Al_2_O_3_ grains. This is the main reason of deteriorate of dynamic compressive strength more than 0.2 wt.% of MgO addition. Therefore, the SHPB test shows the improvement of the dynamic compressive strength only up to a tiny amount (0.2 wt.% of MgO addition) into ZTA ceramics.

## 1. Introduction

Advanced ceramics materials are utilized in many applications such as cutting inserts [1,2,3,4], electrical applications [5] and armor [6,7,8,9] based on their specific properties such as excellent strength, high hardness, good wear resistance and chemical stability [10,11,12]. Generally, ceramics are subjected to dynamic loading in several applications. The adequacy of ceramics’ usage in these applications largely depend on their response under dynamic loading. Unfortunately, failure mechanism of the brittle materials such as ceramics under dynamic loading is not fully understood yet. This is due to the complexity of the mechanical response of ceramics compared to the metals, particularly with respect to the influence of dynamic loading and multiaxial stress states [13]. Consequently, it is a challenging task to utilize new ceramics for armor applications due to the lack of an accurate constitutive model to predict the response of ceramics under the dynamic loading. Therefore, the efforts of designers and scientists have been focused on using other parameters to roughly estimate the dynamic compressive strength of ceramics such as hardness [13,14,15]. Hardness is the most common parameter to determine the compressive strength and dynamic behavior of the new ceramics [16]. Lankford et al. [7,17,18,19] comprehensively studied the dynamic strengths of ceramics and demonstrated that hardness can be used as the upper limit for the dynamic compressive strength. Lankford [7] found that it is possible to attain this limit if the material is sufficiently fine-grained and flaw-free. As well as hardness, other parameters like porosity and fracture toughness also can help to estimate the dynamic strength of ceramics. A lot of research has been done to investigate the effect these parameters have on the strength of different materials [20,21,22,23], however only few studies have been carried out on the ceramic materials.

The previous literature of dynamic behavior of ceramics is mostly limited to few ceramic materials such as Al_2_O_3_ [24], SiC [25], and AlN [26,27,28]. Caccia et al. [29] study the dynamic behavior of the SiC-based composite by using the SHPB (in range of 500–1500 s^−1^). They found the SiC based composite shows the higher strength (around 3×) in high strain rate compare to the quasi static strength. Luo and Chen [24] studied the dynamic response of the intact and damage alumina by the double pulse Split Hopkinson Pressure Bar (SHPB). They found the strength of the damage ceramic after the impact is extensively lower than the intact ceramic, damaged ceramic is not very sensitive to strain rates. The Brazilian test is another method to determine the tensile strength of brittle materials. This method is a typically useful test for indirectly measuring the tensile strength of brittle materials which disc specimen is placed between the incident bar and transmission bar of SHPB. Details of this method could be find in the literature [30]. Chen et al. [30] carried out the dynamic Brazilian test for Al_2_O_3_ sample and coupled it to the digital image correlation to conduct a full-field deformation analysis of the specimens. They found that the dynamic behavior of ceramics was significantly influenced by microstructural characteristics; including phase morphology, grain size and shape, composition and texture. However, the effect of different microstructure on the dynamic behavior of the ceramic is rarely reported. Duan et al. [31] also coupled the 2D digital image correlation (DIC) to the Split-Hopkinson pressure bar (SHPB) to study the impact behavior of materials. Arab et al. [32] reported the effect of different percentages of zirconia (10–40 wt.%) on the ZTA dynamic behavior. They found ZTA with 20% of zirconia has the highest dynamic strength. During the sintering process of ZTA, Zirconia was expended slightly more compared to the alumina, and due to this, expansion of the alumina grain was prohibited by Zirconia grain. As a result, ZTA has a comparable high hardness combined with a relatively fair fracture toughness [33]. Therefore, it can be a promising candidate for applications that are subjected to dynamic loading.

The enhancement of fracture toughness and hardness of ZTA ceramics has received a great amount of interest from many researchers [34,35,36,37]. Thus, appreciable attempts have been made to address effective approaches to improve ZTA ceramics’ mechanical properties through dispersing suitable second phase reinforcements such as whiskers, fibers, ductile particles or platelets within the continuous ZTA matrix. It has been revealed to be a simple and feasible strategy for obtaining enhanced hardness and toughness [38]. For example, dispersing oxides into ZTA matrix to tailor the microstructure of ZTA and consequently, achieving superior mechanical properties of ZTA. In this regard, it was postulated that addition of MgO into Al_2_O_3_-based ceramics reduces the sintering temperature and grain size [39]. Addition of small amounts of MgO (<0.25 wt.%) enabled Al_2_O_3_ to sinter to near theoretical density [40] and it is favorable to inhibit the discontinuous grain growth and to foster the sintering of Al_2_O_3_-based ceramics, leading to completely dense and strongly finer homogeneous structure [41]. The grain size of Al_2_O_3_ decreased thanks to the pinning effect of MgO additive on its microstructure [42]. Azhar et al. [43] observed adding the MgO to the ZTA can improve the hardness. However, there are only a few studies regarding the dynamic behavior for ZTA [32,44]. Therefore, the current work is presented to fill this gap by discussing the mechanical properties such as hardness and fracture toughness of ZTA added with MgO, and their relationship with the dynamic strength and with the dynamic strength was examined.

## 2. Experimental Details

### 2.1. Materials and Sample Fabrication

The starting raw materials are Al_2_O_3_ powder (Martinswerck, Bergheim, Germany, 99 wt.%, average particle size 0.5 µm) was wet mixed with YSZ powder (Goodfellow, Huntingdon, England, ZR616010, 5.4 wt.% Y_2_O_3_ as stabilizer, average particle size 1.5 µm) and MgO powder (Strem Chemicals, Newburyport, MA, USA, 99.95 wt.%, average particle size 0.75). The weighted raw materials are mixed with distilled water using the Penta Power Multi Drive mixer for a duration of 8 h at the speed of 60 rpm. The mixed powder was dried in the oven for 24 h at 100 °C. Then the dried mixture was compact at 300 MPa for 2 min using on hydraulic press. ZTA samples were formed from mixing 4/1 ratio of Al_2_O_3_/YSZ according to the previous work [42]. The ZTA mixture was added with different amounts of MgO (0–0.9 wt.%). The details of preparation procedures can be found elsewhere [34,35,36]. Two different sizes of ZTA pellets were prepared. The cylindrical pellet (10 mm in diameter and 4 mm in thickness) for hardness and fracture toughness tests and smaller cylindrical pellets (4.5 mm in diameter and 6 mm in thickness) for SHPB test were prepared. The ZTA pellets were sintered at 1600 °C for 4 h with 5 °C/min heating and cooling rates.

### 2.2. Characterisation

The phase analysis of the sintered samples was examined through suing an x-ray diffractometer (XRD) diffractometer (Bruker AXS D2 Advance, Billerica, MA, USA) with CuKα radiation operating at 30 kV and 10 mA. Counting time was fixed at 38.4 s and the scanning speed was maintained at 0.03°/s in the range of 10° ≤ 2θ ≤ 90°. Field emission scanning electron microscopy (FESEM Ziess Supra 35 VP, Pleasanton, CA, USA) was utilized to study the microstructure of the samples. Archimedes principle was employed to measure the density and porosity of the samples (ASTM C373-14a [45]). The Vickers Indentation Fracture (VIF) technique was used to obtain the Vickers hardness and fracture toughness of the sintered samples. The hardness and fracture toughness data was obtained through using the hardness tester (Shimadzu Vickers hardness tester HSV-20, Kyoto, Japan) by taking the average of five different readings for each sample. The polished sintered samples were subjected to HV 30 kgf for 10 s. ASTM E384-17 [46] was used for the Vickers hardness test.

The indention was applied by pressing the indenter perpendicular to the surface of the highly polished sample. Using these indention measurements, the fracture toughness of the sample was calculated by equation proposed by Niihara [47].

(1) $$3K_{Ic(HV30)}=0.035(H\times a^{0.5})\times {\left(\frac{3E}{H}\right)}^{0.4}\times {\left(\frac{d}{a}\right)}^{-0.5}$$

where *H* is the Vickers hardness, *a* is the half distance of indent diagonal, *E* is Young modulus and *d* is the crack length.

**Figure 1:**
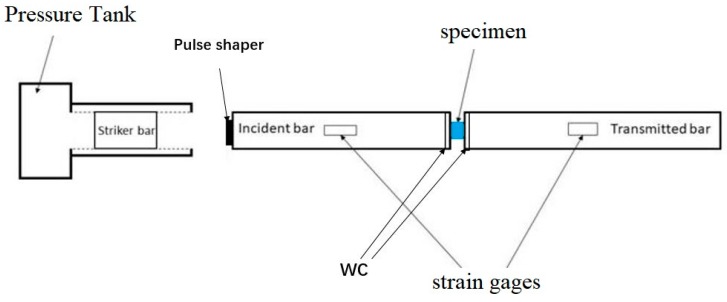
Schematic diagram of the, modified of Split Hopkinson Pressure Bar (SHPB) set up for testing the ceramic.

The dynamic compressive strength of ceramics were measured by using the SHPB modified for testing the ceramics [48,49]. Figure 1 shows the schematic setup of the modified SHPB Traditionally, thin ductile (copper, aluminum) metallic disk is used as pulse shapers to generate the ramp shape pulse. Upon impact by a striker, ramp pulse in the incident bar produces due to the plastic deformation of the ductile metallic disk a. The rise and fall times in the ramp pulse can be controlled by changing the material, diameters and thickness of the pulse shaper as well as the length and velocity of the striker bar. A copper pulse shaper (a thickness of 2 mm and diameter of 10 mm) [50] was located between the striker and incident bars. To prevent the ceramics samples from indenting the incident and transmission bars faces, tungsten carbide platen disk were placed between the samples and bars face. These platens have the same impedance with bars. After each test the surface of the Tungsten Carbide (WC) disc is examined by the optical microscope to make sure no cracks or other damage is occurred during the test. These observations reveal that no cracks or damage occurred during the test for the WC disc. If the failure of WC discs occurs, the ceramic fractures invariably (due to stress concentration caused by the WC discs fragments on the ceramic), thus rendering the data on ceramic fracture strength invalid. Both incident and transmission bars are made of SUJ2 with a diameter of 12 mm and length of 150 cm. The pressure of the gas gun tank is fixed at 0.35 MPa. Reflected and transmitted pulses were recorded by the strain gauges placed on the incident and transmitted bars respectively. The stress, strain and strain rate of the samples were calculated using the standard procedures.

The stress (*σ_s_*), strain (*ε_s_*) and strain rate (${\dot{\varepsilon }}_{s}$) in the specimens were calculated using the following equations:

(2) $$\sigma_{s}\left(t\right)=-E_{b}\frac{A_{b}}{A_{S}}\varepsilon_{t}\left(t\right)$$

(3) $$\varepsilon_{s}=2\frac{C_{0}}{L_{s}}\int_{0}^{t}\varepsilon_{r}\left(t\right)dt$$

(4) $${\dot{\varepsilon }}_{s}=2\frac{C_{0}}{L_{S}}\varepsilon_{r}(t)$$

where subscript *b* is referred to the bar and *s* is referred to sample, *A* is the cross section area and *L* is length, *C_o_* is $\sqrt{\frac{E}{\rho }}$, *E* is Young modulus of the bar and  ρ  is bar density, $\varepsilon_{t}$ is the transmitted strain and $\varepsilon_{r}\;$ is reflected strain.

## 3. Results and Discussions

**Figure 2:**
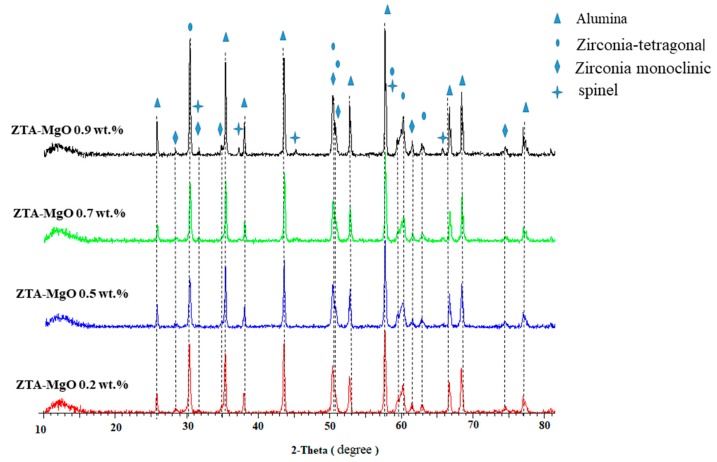
XRD patterns of ZTA-MgO samples sintered at 1600 °C for 4 h.

Figure 2 shows the XRD of ZTA-MgO samples sintered at 1600 °C for 4 h. The XRD patterns show the presence of four phases in these samples, including Al_2_O_3_ in corundum phase (ICDD no. 00-046-1212), YSZ (tetragonal phase) (Zr_0.935_Y_0.065_O_1.968_) (ICDD no. 01-078-1808), baddeleyite (monoclinic phase or m-ZrO_2_) (ICDD no. 00-037-1484) and magnesium aluminum oxide or spinel (MgAl_2_O_4_) (ICDD no. 01-073-1959). This is in line with the previous work by Azhar et al. [42] where Al_2_O_3_ and with small amount of the MgO forming a solid solution. The MgO phase was not observed in the XRD pattern of the samples. However, by increasing the MgO concentration and reaching the solid solubility limit, MgO and Al_2_O_3_ can react and form spinel (MgAl_2_O_4_). Thanks to the restructured atoms arrangement in crystal structure, spinel formation is accompanied by 5–7% volume expansion which it results in low sinterability of the prepared material.

**Figure 3:**
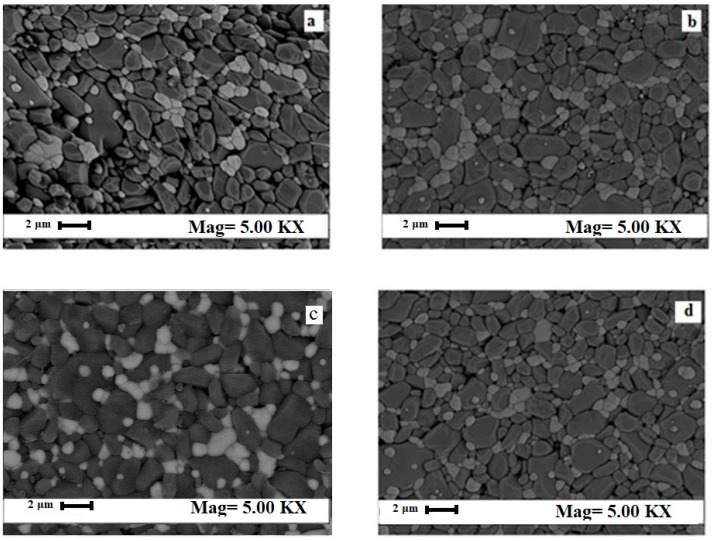
Microstructure of ZTA-MgO samples. (**a**) 0.2 wt.% MgO, (**b**) 0.5 wt.% MgO, (**c**) 0.7 wt.% MgO, and (**d**) 0.9 wt.% MgO.

The phase compositions were confirmed by FESEM image of ZTA-MgO samples (Figure 3) which shows the Al_2_O_3_ grain size decreases with increase of MgO addition. The decrease of Al_2_O_3_ grain size was due to the pinning effect of MgO [42]. The MgO inhabited the grain growth of Al_2_O_3_. This mechanism can be explained through (i) grain boundary mobility reduction; (ii) increasing the pore mobility due to the increment of surface diffusivity; (iii) increasing densification rate through promoting lattice and boundary diffusions and (iv) decreasing the grain boundary anisotropy and surface energy of grains [41]. This is in line with the findings in the current study. FESEM micrographs show smaller Al_2_O_3_ grain with MgO addition up to 0.7 wt.%. Nevertheless, addition of MgO more than the aforementioned ratio lost the beneficial effect of MgO addition which shows larger grain size of Al_2_O_3_. Concurrently, addition of MgO positively affects porosity which removes more pores from the ZTA matrix as shown in FESEM micrographs (Figure 3). Therefore, the porosity of the sample decreases with smaller grain size in the microstructure.

**Figure 4:**
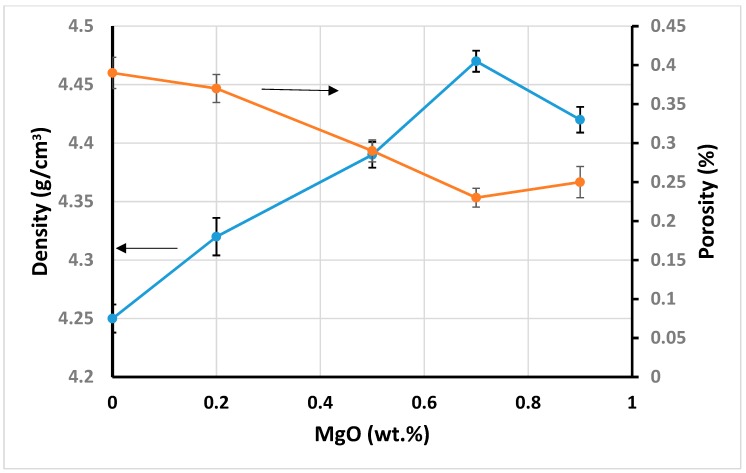
Effects of MgO on density and porosity of samples.

These findings are supported by Figure 4 for bulk density and porosity of ZTA-MgO samples. The more addition of MgO to ZTA, the denser ZTA samples (up to 0.7 wt.% of MgO addition). Oppositely, the porosity of ZTA samples decreases with adding the MgO. Nonetheless, excessive addition of MgO into the ZTA shows an opposite trend of density and porosity behavior due to less effective pinning effect of MgO. The density increased from 4.25 g/cm^3^ to the 4.47 g/cm^3^ at 0.7 wt.% MgO, showing an increase of 5.1% in density. A minimum porosity of 0.22% was observed for 0.7 wt.% MgO. These findings were further supported by the outcome of previous works by Azhar et al. [42].

**Figure 5:**
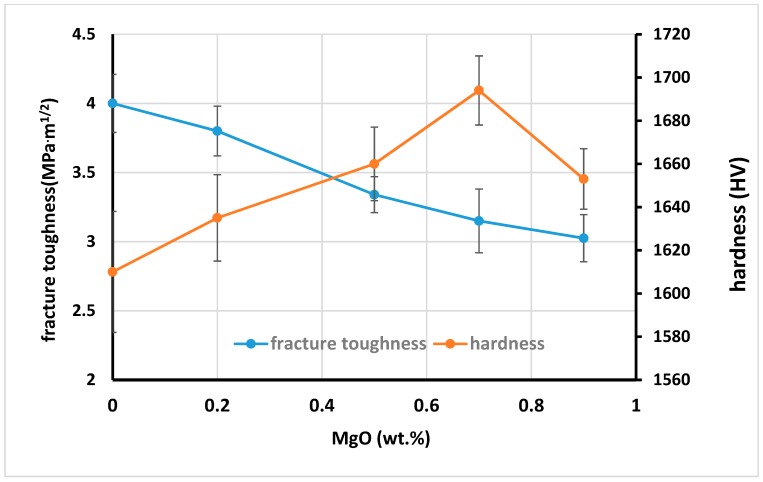
Effects of MgO addition in Zirconia toughened alumina (ZTA) on fracture toughness and hardness.

**Figure 6:**
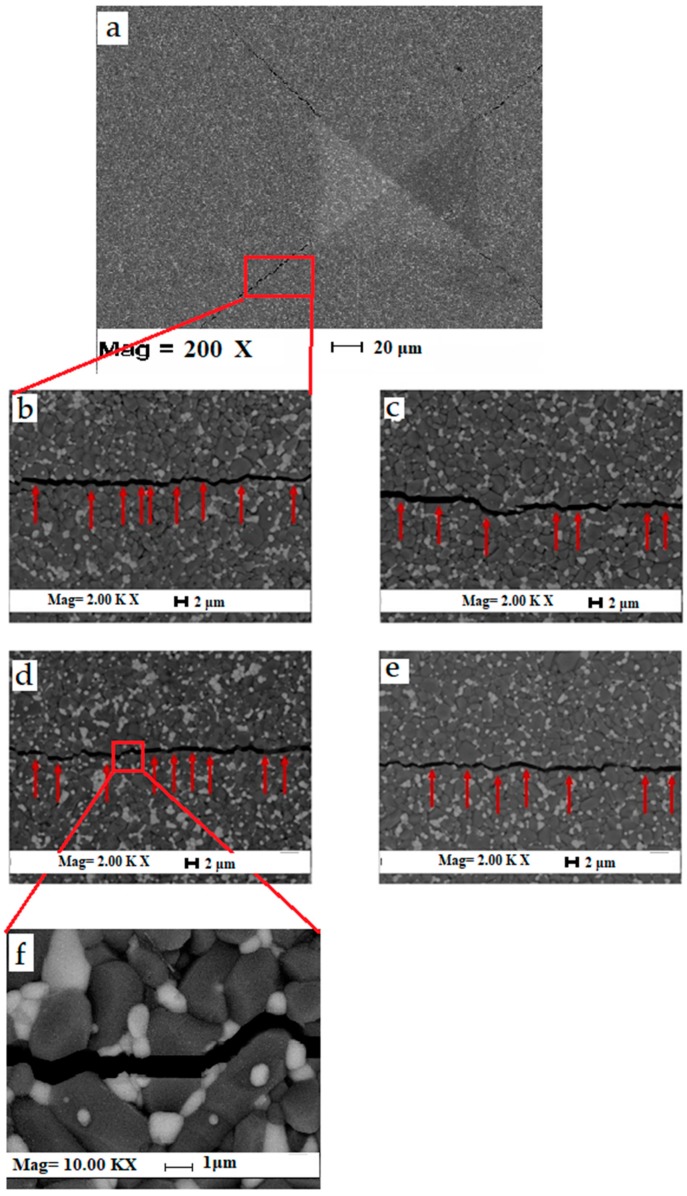
Crack propagation of ZTA-MgO samples. (**a**) Cracks at tips of Vickers hardness indent tips (**b**) 0.2 wt.% MgO, (**c**) 0.5 wt.% MgO, (**d**) 0.7 wt.% MgO, (**e**) 0.9 wt.% MgO and (**f**) high magnification of crack.

Figure 5 shows the fracture toughness and Vickers hardness of ZTA-MgO samples with different MgO addition. The fracture toughness of ZTA samples was reduced with the increase of MgO addition (Figure 5). The fracture toughness is decreased from 4 MPa·m^1/2^ for pure ZTA to 3.02 MPa·m^1/2^ for ZTA added with 0.9 wt.% MgO. The decrease of fracture toughness is affected by the small grain size of ZTA samples with 0–0.7 wt.% MgO addition and a slight increase in porosity afterwards. Smaller grain size reduces the toughness due to decreased load bridging capability of smaller grain bridges [42]. The previous work of Rittidech et al. [39] shows addition of MgO led to decrease of the fracture toughness significantly, which is in agreement with the present research. Smaller grain size results in lower intrinsic toughness due to reduced load bridging capability of smaller grain bridges. Riu et al. investigated that the increase of Al_2_O_3_ grains sizes due to crack bridging by large platelike grains led to enhancement of the fracture toughness and flaw tolerance. Additionally, increase in porosity with more addition than 0.7 wt.% led to further decline in fracture toughness. Moreover, with more addition of MgO into ZTA, intergranular fracture mode is increased. It was postulated that transgranular fracture mode consumes more energy compared with the intergranular fracture [33]. Therefore, due to the increase of the intrgranular fracture, the fracture toughness of the sample is decreased. Figure 6 shows both intergranular and transgranular fracture modes for ZTA-MgO samples, the red arrow shows the transgranular fracture mode. For summary of this part properties of ZTA with different amount MgO is listed in the Table 1.

However, the hardness of ZTA-MgO ceramics has an opposite trend. The hardness increases with the increase of MgO addition varying from 1610 HV for pure ZTA to 1694 HV for ZTA added with 0.7 wt.% MgO addition. The improvement of Vickers hardness is governed by the reduction of grain size of the Al_2_O_3_, which resulted from the microstructure pinning effect due to the MgO addition. Nonetheless, further addition of 0.7 wt.% MgO reduced the hardness. The reason is related to the formation of a new phase for ZTA samples containing more than 0.7 wt.% MgO as shown in XRD patterns (Figure 3) [51,52]. Therefore, further addition of the MgO cannot delay the grain growth of Al_2_O_3_. Additionally, the porosity of ZTA samples increased with an addition of 0.7 wt.% of MgO as shown in Figure 4 for bulk density and porosity of ZTA samples. Moreover, the hardness of MgAl_2_O_4_ is lower (around 1410 HV) compared to the Al_2_O_3_ hardness.

**Figure 7:**
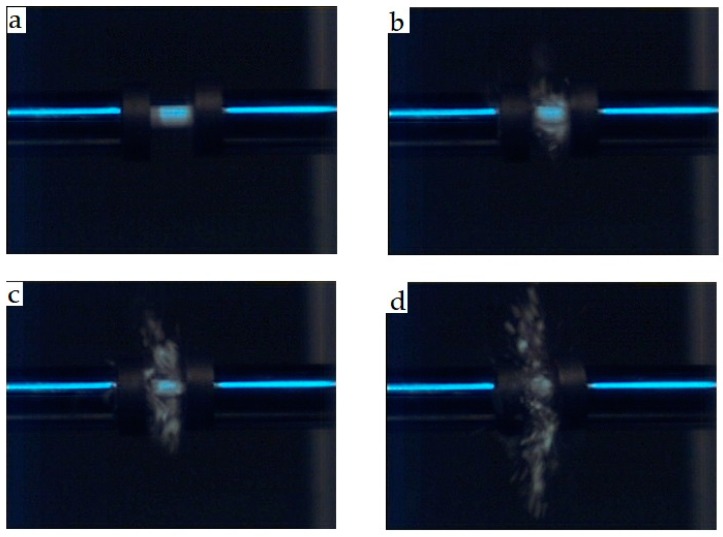
Failure of sample during the dynamic loading. (**a**) 0 µs; (**b**) 50 µs; (**c**) 100 µs; (**d**) 150 µs.

**Figure 8:**
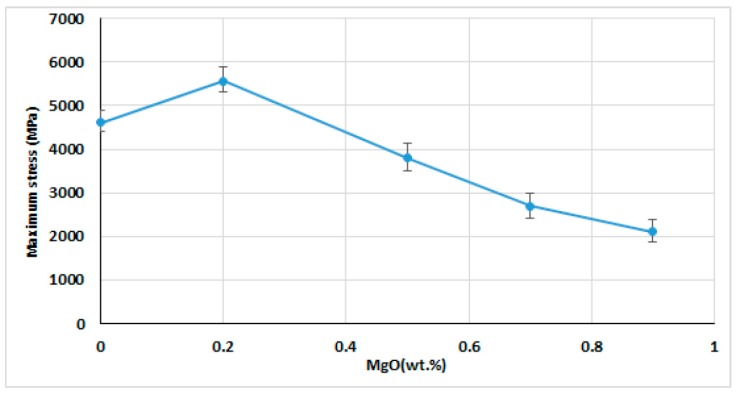
Maximum dynamic compressive stress of ZTA added with different amount of MgO.

During the dynamic test by the SHPB, failure occurs for all samples (Figure 7). The sample’s failure for ZTA with 0.2 wt.% MgO during the dynamic loading captured by high-speed camera is shown in Figure 7. Figure 7a shows the intact sample before applying the dynamic load. After applying the dynamic load, cracks initiated from the contact surface between the bars and sample (Figure 7b,c), cracks propagated into the inner surface of the sample, with a tiny crack being the source of crack growth nucleation. The cracks in the network coalesced to form a larger damage area, leading to complete damage Figure 7d. Multiple specimens are tested for each sample. The maximum stress on each specimen’s stress-strain curve is taken as the dynamic compressive strength, prior to the maximum stress, crack that began from the contact surface between the sample and bars. As the compressive loading proceeds, cracks propagated across the sample (along the loading direction), these cracks coalesce, the ceramic begins to lose its load-carrying capacity, and the stress collapses and massive failure ensues. Figure 8 shows dynamic compressive failure stress of ZTA with different amounts of the MgO, indicating that the sample with 0.2 wt.% MgO exhibits the highest dynamic compressive failure stress among all samples. Dynamic compressive failure stress of the sample with 0.2 wt.% MgO shows an improvement of 16% compared with the pure ZTA sample. However, it was observed that adding more MgO into ZTA causes decrease of the dynamic compressive failure stress of ZTA composites. Dynamic strength of ceramic is raised by increasing the strain rate, however, in the current work, the strain rate was kept constant (around the 3500 s^−1^) in all tests.

The dynamic compressive failure stress is directly influenced by the fracture toughness, hardness and porosity for the samples added with different amount of MgO. Usually, cracks are nucleated by porosities in the sample matrix. Hence, the lower porosity, the lower the nucleation of cracks. Although the samples’ porosity is reduced for addition up to 0.7 wt.% MgO, dynamic compressive failure stress improvement is only limited up to 0.2 wt.% MgO. This is due to the fracture toughness which reduces with more MgO addition. It is well known that wing cracks grew longer with lower fracture toughness. These longer wing cracks have increased probability for the cracks to meet each other to form bigger wing cracks. Coalescence of the cracks network led to forming a bigger damage area and therefore, the samples were completely damaged. It should be mentioned that experiments carried out under compressive loading actually fail during tension. The global compressive stress field is changed to the localized tensile stress field around the microcracks and defects.

A sample added with 0.2 wt.% MgO has the highest value of the dynamic compressive failure stress. This feature can be explained due to its higher fracture toughness since higher stress is required to activate the preexisting microcracks.

**Figure 9:**
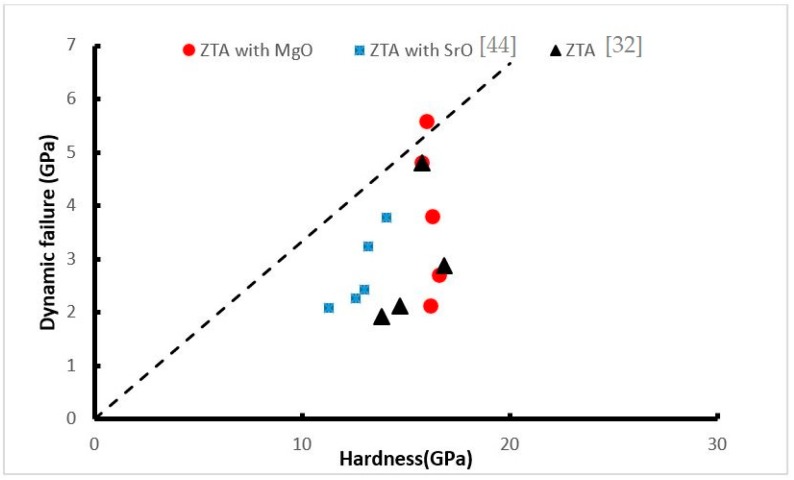
Relationship between the hardness and dynamic strength.

The results of the relationship between the hardness and dynamic failure stress in the current work and previous work [32,44] is illustrated in Figure 9. All of these data except that of ZTA with 0.2 wt.% MgO are below the line suggested by Lankford et al. [53] (Failure stress = (Hardness/3)). It is shown that the Lankford line could not be the upper limit for the dynamic failure stress of the ceramic. Lankford et al. [53] equation (H/3) assumed that the third of pressure is shear stress (deviatoric) and the remainder beneath the indenter is hydrostatic. They believed this limit can be attained around Hugoniot elastic limit or in the case the sample has the fine grain or be the free flaws. In reality, the actual uniaxial strength of ceramic rarely reached this amount due to the coalescence and microcracking that could cause failure prior to the occurrence of plastic flow. Nevertheless, in the current research; ZTA with 0.2 wt.% MgO reached to this limit in strain rate around the 3500 s^−1^. By adding the 0.2 wt.% MgO to the ZTA, the grain size decreases as well as porosity that lead to increasing the dynamic failure strength of the sample and it is being closer to the upper limit of the dynamic compressive failure stress. The dynamic compressive failure stress of the ceramic is dependent on many parameters such as grain size, porosity and density of microcracks [32]. During the compressive loading, frictional sliding along the flaw faces leads to the development of wing cracks at the tips of the pre-existing flaws, starting with flaws having the largest size. The wing cracks grow parallel to the direction of the applying compressive load. The close wings cracks meet each other and formed the bigger cracks. By increasing the density of the flaws and crack, the strength of the ceramic decrease dramatically [44]. By increasing the density of the flaws, pores and microcrack the strength of the ceramic decreases dramatically [44].

**Figure 10:**
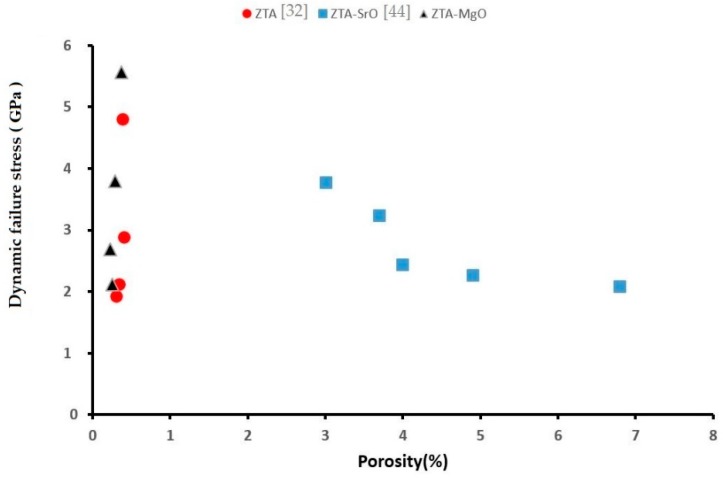
Relation between the porosity and dynamic failure.

As mentioned above, the porosity of ZTA samples decreases with adding the MgO. Chen et al. suggested the use of Equation (5) for porous alumina and zirconia samples:

(5) $$\sigma_{c}=\sigma_{0}e^{-kP}$$

where *σ_c_* is the compressive strength of the sample, *σ*_0_ is the compressive strength of sample without porosity, *k* is an empirical constant, and *P* is the porosity. Based on this equation, decreasing the porosity in the sample leads to increase of the compressive strength. Figure 10 shows the relationship between the porosity and dynamic failure stress in the current work and our previous works [32,44].

This result shows the porosity of sample does not have a direct effect on the sample dynamic failure. However, increasing the porosity can lead to decreasing the strength of the sample due to the higher probability of crack nucleation during loading. When the sample is subjected to compressive loading, frictional sliding along the flaw faces leads to the development of wing cracks at the tips of the pre-existing flaws, starting with flaws with the biggest size. The wing cracks grow parallel to the direction of the applying compressive load. The close wings cracks meet each other and form the bigger cracks. In this graph the ZTA-SrO shows the different behavior compared to the other samples, this is different behavior is due to the high porosity of this sample, to produce the ZTA-SrO, SrCO_3_ is used as additive. The addition of SrCO_3_ to the ZTA increased the number of pores in the samples, pore formation is related to the decomposition of SrCO_3_, SrO, and CO_2_ during the sintering process. These pores could act as nucleation sites for cracks when subjected to dynamic compressive load. Also, by adding the SrCO_3_ to the ZTA, strontium aluminum oxide (SrAl_12_O_19_) is formed in the ceramic, and it changes the crack propagation mechanism and decreases the strength of ZTA.

## 4. Conclusions

The addition of MgO into ZTA has various effects on the microstructure, phase formation and mechanical properties of ZTA. The microstructure of ZTA-MgO samples observed less porosity with more addition of MgO with denser ZTA samples. Additionally, spinel was formed with addition ≥0.7 wt.% MgO. The combination effect of these features was reflected in the positive effect on the hardness improvement while decreasing the fracture toughness of ZTA ceramics. This is due to pinning effect of MgO, reducing the grain size of Al_2_O_3_ and reducing the porosity. Therefore, the dynamic compressive strength is expected to increase. However, the ZTA added with 0.2 wt.% MgO has the highest compressive strength compared to other samples. The reason for this is attributed to the continuous decrease of fracture toughness of ZTA samples with more MgO addition.

## Figures and Tables

**Table 1:** Properties of ZTA with different amount MgO.

Name	Density (gr/cm^3^)	Hardness (HV)	Fracture Toughness (MPa·m^1/2^)
ZTA-0.2 wt.% MgO	4.32	1635	3.8
ZTA-0.5 wt.% MgO	4.39	1660	3.34
ZTA-0.7 wt.% MgO	4.47	1694	3.15
ZTA-0.9 wt.% MgO	4.42	1653	3.02
